# A high-salt diet enhances leukocyte adhesion in association with kidney injury in young dahl salt-sensitive rats

**DOI:** 10.1038/hr.2017.31

**Published:** 2017-03-16

**Authors:** Hidenori Takahashi, Suguru Nakagawa, Yaqiong Wu, Yukari Kawabata, Atsushi Numabe, Yasuo Yanagi, Yasuhiro Tamaki, Yoshio Uehara, Makoto Araie

**Affiliations:** 1 Department of Ophthalmology, University of Tokyo School of Medicine, Tokyo, Japan; 2 Department of Ophthalmology, Jichi Medical University, Shimotsuke, Japan; 3 Department of Cardiology, Fourth Hospital of Hebei Medical University, Shijiazhuang, China; 4 Division of Clinical Nutrition, Faculty of Home Economics, Kyoritsu Women’s University, Tokyo, Japan; 5 Department of Medicine, Moka Hospital, Moka, Japan; 6 Medical Retina, Singapore National Eye Centre, Singapore, Singapore; 7 Medical Retina, Singapore Eye Research Institute, Singapore, Singapore; 8 The Ophthalmology & Visual Sciences Academic Clinical Program, Duke-NUS Medical School, National University of Singapore, Singapore, Singapore

**Keywords:** Dahl salt-sensitive rats, kidney injury, leukocyte adhesion molecules, leukocytes, salt-sensitive hypertension

## Abstract

Salt-sensitive hypertension is associated with severe organ damage. Generating oxygen radicals is an integral component of salt-induced kidney damage, and activated leukocytes are important in oxygen radical biosynthesis. We hypothesized that a high-salt diet causes the upregulation of immune-related mechanisms, thereby contributing to the susceptibility of Dahl salt-sensitive rats to hypertensive kidney damage. For verifying the hypothesis, we investigated leukocytes adhering to retinal vessels when Dahl salt-sensitive rats were challenged with a high-salt (8% NaCl) diet using acridine orange fluoroscopy and a scanning laser ophthalmoscope. The high-salt diet increased leukocyte adhesion after 3 days and was associated with a significant increase in mRNA biosynthesis of monocyte chemotactic protein-1 and intercellular adhesion molecule-1 (ICAM-1) -related molecules in the kidney. Losartan treatment did not affect increased leukocyte adhesion during the early, pre-hypertensive phase of high salt loading; however, losartan attenuated the adhesion of leukocytes during the hypertensive stage. Moreover, the inhibition of leukocyte adhesion in the pre-hypertensive stage by anti-CD18 antibodies decreased tethering of leukocytes and was associated with the attenuation of functional and morphological kidney damage without affecting blood pressure elevation. In conclusion, a high-salt challenge rapidly increased leukocyte adhesion through the over-expression of ICAM-1. Increased leukocyte adhesion in the pre-hypertensive stage is responsible for subsequent kidney damage in Dahl salt-sensitive rats. Immune system involvement may be a key component that initiates kidney damage in a genetic model of salt-induced hypertension.

## Introduction

An increasing number of studies suggest that proinflammatory cytokines released from anchored leukocytes have an important role in the progression of cardiovascular damage in metabolic and hypertensive diseases.^1^ The proinflammatory process is an integral component of insulin resistance as well as glucose metabolism.^2^

In spontaneously hypertensive rats (SHRs), monocyte adhesion in association with the expression of intercellular adhesion molecule-1 (ICAM-1) is increased and the adhesion mechanism in the brain microvasculature mediates the onset of spontaneous hypertension (SHR) and organ damage.^3, 4, 5, 6, 7^ Moreover, lipopolysaccharide-stimulated biosynthesis of monocyte chemotactic protein-1 (MCP-1) and ICAM-1/macrophage adhesion ligand-1, a CD-18 receptor and an integral cell-surface protein expressed in most leukocytes, is greater in SHRs than in Wistar Kyoto rats.^8^ The adhered leukocytes are less able to suppress immune mechanisms in SHRs.^9^

Such abnormality in leukocyte–endothelial cell interaction may be mediated by angiotensin II as leukocyte adhesion to the vascular wall is significantly increased in double transgenic rats harboring human renin and angiotensinogen genes.^10^ The activation of nicotinamide adenine dinucleotide phosphate oxidase by angiotensin II produces oxygen radicals. The oxygen radicals increase leukocyte adhesion through transcription factor nuclear factor kappa B and increased synthesis of the adhesion molecule MCP-1.^11^ The renin–angiotensin system/oxygen radicals may be responsible for increasing leukocyte adhesion in SHRs.

Dahl salt-sensitive (Dahl S) rats are a genetic model of salt-induced hypertension in humans. It is well known that Dahl S rats are prone to hypertensive organ damage (that is, cerebral stroke, heart failure and kidney impairment).^12, 13^ Dahl S rats are susceptible to angiotensin II- or oxygen stress-mediated organ damage.^14, 15, 16^ Moreover, it has been reported that leukocyte–endothelial cell interaction is involved in organ damage through endothelin receptor in salt-dependent hypertension in DOCA-salt rats.^17^ Based on these studies, this susceptibility presumably results from the leukocyte adhesion mechanism. In established salt-dependent hypertension of Dahl S rats, the monocyte–endothelial interaction is increased and L-arginine administration restores the capacity for nitric oxide biosynthesis with attenuation of leukocyte adhesion.^18^ These processes strongly suggest that leukocyte–endothelial adhesion is involved in the initiation of hypertension in Dahl S rats.

A method was recently introduced to detect *in vivo* leukocyte–endothelial cell adherence by using retinal vessels and a scanning laser ophthalmoscope.^19, 20^ Real-time tethering and blocking by specific antibodies against adhesion molecules can be monitored. Using such methods in the present study, we examined the influence of a high-salt challenge on leukocyte–endothelial adhesion and assessed the involvement of angiotensin II in the association between high salt intake and leukocyte adhesion. In addition, we directly blocked leukocyte adhesion using anti-ICAM-1 antibodies to assess the pathophysiological implications of the leukocyte–endothelial interaction in salt-induced hypertension and kidney damage in Dahl S rats.

## Methods

### Effect of high salt loading on leukocyte adhesion (experiment 1)

Dahl S/Jr Sea rats utilized in the present study were selectively bred for their blood pressure response to a diet high in salt. This strain was originally obtained from Möllegård (Ejby, Denmark), shared with the Seiwa Animal Laboratory (Fukuoka, Japan), and then maintained as an inbred strain at the Kyudo Laboratory (Kyudo, Saga, Japan).

Sixty 4-week-old male Dahl S rats were divided into two groups: (1) 30 rats were fed a low-salt (0.3% NaCl, w/w) diet (low-salt group); and (2) 30 were fed a high-salt (8% NaCl, w/w) diet (high-salt group). Water was provided *ad libitum*. We evaluated leukocyte adhesion on day 3 after salt loading and then every week throughout the study.

To assess leukocyte adhesion, the rats were anesthetized with an intraperitoneal injection (5 ml kg^−1^) of a mixture (7:1) of 10 mg ml^−1^ ketamine hydrochloride (Ketalar; Sankyo, Tokyo, Japan) and 23 mg ml^−1^ xylazine hydrochloride (Celactal; Bayer, Tokyo, Japan), and each pupil was dilated with one drop of 0.5% tropicamide (Mydrin M; Santen Pharmaceutical, Osaka, Japan). Leukocyte adhesion to the retinal vessels was evaluated using the acridine orange fluorescence method. Three weeks after salt loading (rats 7 weeks of age), the kidneys were processed to evaluate the expression of adhesion molecule mRNA by real-time PCR.

Rats used to investigate leukocyte adhesion were not used for other experiments, but killed after the test period. All rats were used solely for assessment of leukocyte adhesion, immunological characterization of adherent leukocytes or determination of mRNA in the kidney study.

To characterize leukocyte adhesion after salt loading in Dahl S rats prone to salt-induced hypertension, we investigated the adherence of leukocytes in response to the high-salt challenge in a genetic rat model of SHR. Eighteen male SHRs were divided into three groups, and leukocyte adhesion was evaluated in (1) six 4-week-old rats fed a regular (0.75% NaCl, w/w) chow after weaning, (2) six 5-week-old rats fed a low-salt diet for 1 week and (3) six 5-week-old rats fed a high-salt diet for 1 week.

### Effects of angiotensin II receptor blockade on leukocyte adhesion (experiment 2)

Twenty-five 4-week-old male Dahl S rats were divided into the following five groups (*n*=5 per group): (1) rats fed a low-salt (0.3% NaCl, w/w) diet (0.3% DS); (2) rats fed a high-salt (8% NaCl, w/w) diet (8% DS-control); (3) rats fed a high-salt diet and treated with losartan (30 mg  per kg body weight [BW] per day) for the first 10 days (8% DS-early); (4) rats fed a high-salt diet and treated with losartan (30 mg per kg BW per day) for the last 10 days (8% DS-late); and (5) rats fed a high-salt diet and treated with losartan (30 mg per kg BW per day) throughout the experiment (8% DS-whole). Water was available *ad libitum* during the experiment. The rats were maintained for 20 days; at the end of the experiment, the rats were anesthetized and the leukocytes adhering to the retinal vessels were detected. The mRNA levels of the adhesion molecules in the kidney were measured using real-time PCR.

### Effects of adhesion blockade with anti-CD18 antibodies on kidney damage (experiment 3)

To elucidate the pathophysiological role of leukocyte adhesion in the initiation of kidney damage in Dahl S rats, we blocked adhesion using an anti-CD18 (integrin β-2) antibody, the integral cell-surface proteins involved in leukocyte adhesion. Thirty-six 4-week-old male Dahl S rats were divided into three groups (*n*=12 per group): (1) rats fed a low-salt (0.3% NaCl, w/w) diet and injected with nonspecific mouse IgG (1 mg kg^−1^; Southern Biotech, Birmingham, AL, USA) as the control antibody (control [LS]), (2) rats fed a high-salt (8% NaCl, w/w) diet and injected with the control antibody (HS-IgG), and (3) rats fed a high-salt (8% NaCl) diet and injected with mouse anti-rat CD18-specific antibodies (1 mg kg^−1^ BW) (clone WT.3; Serotec, Oxford, UK) (HS-anti-CD18). The antibodies were injected intraperitoneally every 2 days for the first 10 days.^21, 22^ The rats were killed at week 1 or 3 to evaluate leukocyte adhesion and inhibition of binding to the retinal vessels in animals challenged with a high-salt diet.

Systolic blood pressure was measured using the tail cuff method, with a modified detection system (Natsume Seisakujo Model KN-210-1; Tokyo, Japan).^23, 24^ The same investigator measured the blood pressure of all rats in a quiet, warm room. The rats were placed in metabolic cages to collect 24- h urine samples weekly to determine the variables that indicate kidney damage. The urine samples were stored at −80 °C until assayed.

At week 1 and at the end of the study, blood samples and organs of interest were obtained under anesthesia. Retinal vessels were perfused with 250 ml kg^−1^ BW phosphate-buffered saline containing 1.25 mg kg^−1^ BW fluorescein isothiocyanate-conjugated concanavalin A (ConA) lectin (Vector Laboratories, Burlingame, CA, USA), as described previously.^19^ Both eyeballs were enucleated to determine leukocyte adhesion to the retinal vessels using stained retinal flat mounts.^19, 20^ The separated plasma samples were stored at −80 °C until assayed. The weights of the kidney and heart were standardized relative to body weight. A portion of the kidneys was placed in 10% formalin solution for morphological examination.

### Acridine orange fluorography for *in vivo* determination of adherent leukocytes

Leukocyte adhesion to the retinal vessels was determined using acridine orange fluorography.^25^ A scanning laser ophthalmoscope (Rodenstock Instruments, Munich, Germany) was used to obtain interlaced video frames of the fundus stained using metachromatic fluorochrome acridine orange (Wako Pure Chemicals, Osaka, Japan). The dye emits green fluorescence when interacting with DNA. The maximum excitation and emission wavelengths of the acridine orange–DNA complex are 502 and 522 nm, respectively. An argon blue laser was used for excitation, with a regular emission filter for fluorescent angiography.

The laser focus was adjusted to the deep retinal capillary layer. Fluorescent leukocytes were recorded for 10 s on a DVCPRO digital videotape at 30 frames per  second, with two interlaced fields per frame. The recorded videos were transferred to a computer system equipped with ImageJ software (National Institutes of Health, Bethesda, MD, USA). The recorded video frames were stacked to one frame to enhance arrested leukocyte signals and depress noise from flowing and rolling leukocytes. All leukocytes adhering to the retina around the optic disk (5-disk diameter) were counted. A 10° × 10° angle of view of the retina (170 × 170 pixels) was used for leukocyte detection.

### Immunological characterization of adherent leukocytes

Leukocytes adhering to the retinal vessels were identified using retinal flat mounts and anti-CD18 antibodies. Briefly, retinal vessels were perfused with 250 ml kg^−1^ BW phosphate-buffered saline containing 1.25 mg kg^−1^ BW fluorescein isothiocyanate-conjugated ConA lectin, as described previously.^18^ The eyes were enucleated and immediately placed in 4% paraformaldehyde. Next, the anterior segment was removed and the retina was carefully dissected from the eye cup.

Four cuts were made from the edge to the equator of the retina. Flat mounts were permeabilized using 1% Triton X (Sigma, St. Louis, MO, USA) for 8 h; nonspecific binding was blocked using 5% goat serum. The retinal samples were then incubated for 8 h at 4 °C in the presence of mouse anti-rat CD18 antibodies (dilution, 1:100; clone WT.3; Serotec). After washing with phosphate-buffered saline, the retinal samples were incubated with Alexa Fluor 594-conjugated anti-mouse immunoglobulin goat antibodies (dilution, 1:300; Alexa Fluor 594, A11032; Invitrogen, Carlsbad, CA, USA) at 25 °C for 1 h. The retinal samples were flat mounted with the outer segment facing down using mounting medium for preserving fluorescence (Vectashield; Vector Laboratories), and the samples were examined under a fluorescence microscope (Olympus BX51; Olympus, Tokyo, Japan) at 40 × magnification.^19^ Because capillary vessels infiltrate deep into the retina, making it difficult to detect adherent leukocytes, we detected the leukocytes adhering to the arterioles and smaller veins. Images were acquired using a charge-coupled device camera and imported to a computer. We also used mouse anti-rat CD11a antibodies (dilution, 1:100; WT.1; ab21340-100; Abcam, Cambridge, UK), mouse anti-rat CD11b antibodies (dilution, 1:100; ab8879-100; Abcam), and mouse anti-rat CD45 antibodies (dilution, 1:100; 550566; BD Pharmingen, San Diego, CA, USA) for studying the adherent leukocyte profiles.

### Detection of adhesion molecule mRNA in the kidney

The deep cortex (20 mg) of kidney samples frozen in liquid nitrogen and stored at −80 °C was rapidly homogenized at −4 °C using a MagNA Lyser (Roche Diagnostics, Mannheim, Germany) at 6500 rpm for 50 s. Total RNA was extracted from each tissue using the High Pure Tissue/Isolation Kit (Roche Diagnostics) according to the manufacturer’s instructions. Total RNA concentrations and purity were determined using an ND-1000 spectrophotometer (Thermo Fisher Scientific, Waltham, MA, USA).

Highly purified RNA (460 μg) was used for cDNA synthesis with a Transcriptor First Strand cDNA Synthesis Kit (Roche Diagnostics) according to the manufacturer’s protocol. Real-time PCR was performed using a LightCycler TaqMan Master and a LightCycler ST300 system (Roche Diagnostics). Primers were designed using ProbeFinder software (http://qpcr.probefinder.com/organism.jsp). Probes were selected from Universal ProbeLibrary probes 1–165 for LightCycler (Roche Diagnostics) (Supplementary Table 1). The PCR reaction mixture consisted of 4 μl of 5 × LightCycler TaqMan Master mix, 200 nm forward and reverse primers, 100 nm of the specific Universal ProbeLibrary probe, and 5 μl of the cDNA template. The total volume was adjusted to 20 μl using PCR-grade distilled water. Amplification conditions were set according to the manufacturer's instructions.

### Evaluation of kidney damage

Glomerular sclerosis was semi-quantitatively assessed, according to a previously reported method.^12, 13, 14, 26^ Briefly, each kidney was immediately fixed in a 3.5% formalin solution, and sagittal slices were cut and embedded in paraffin. Sections (thickness, 2 μm) were cut and stained using Azan and periodic acid-Schiff stains. At least 100 glomeruli were examined in each specimen. Glomerular sclerotic lesions were intensely stained using periodic acid-Schiff. Lesion severity was graded according to the percentage of total glomeruli involved as follows: stage 0, no lesions; stage 1, 1–25% sclerosis; stage 2, 26–50% sclerosis; stage 3, 51–75% sclerosis; and stage 4, 76–100% sclerosis. An overall glomerular sclerosis score was calculated by multiplying the severity score (0–4) by the percentage of glomeruli affected and obtaining the total of these values.

Plasma and urinary creatinine levels and urinary sodium excretion were measured using an autoanalyzer (Model Hitachi 736; Hitachi, Tokyo, Japan). Urinary protein concentrations were measured using a protein assay kit (BioRad, Hercules, CA, USA).

### Statistical analysis

Values are expressed as the mean±s.d. Differences were assessed using the Kruskal–Wallis test followed by the Mann–Whitney U-test with Bonferroni correction for non-parametric distribution and one-way analysis of variance followed by *post-hoc* analysis using the Scheffe test for parametric distribution. Correlation was performed using simple Pearson correlations. Multiple regression analysis was performed using maximum likelihood estimation with the Akaike information criterion. Statistical analysis was performed using JMP software (SAS Institute, Cary, NC, USA). *P*-values <0.05 were considered statistically significant. Correlation and multiple regression analyses were performed for 8% salt-loading rats.

### Guidelines for animal experiments

All animal experiments were performed according to the guidelines of the Association for Research in Vision and Ophthalmology and were approved by the Animal Care Committee of the Tokyo University Hospital. The experiments were conducted in accordance with the National Institutes of Health guidelines.

## Results

### High-salt diet increases leukocyte adhesion in young Dahl S rats

We assessed changes in leukocyte adhesion to retinal vessels by acridine orange fluoroscopy when Dahl S rats were challenged with a high-salt diet. On day 3, the high-salt group showed higher systolic blood pressure than the low-salt group (125±2 mm Hg (*n*=19) *vs.* 123±2 mm Hg (*n*=18), *P*<0.05); however, the blood pressure level was within the normal range. Blood pressure increased in a time-dependent manner in both the high-salt and the low-salt groups (Table 1). The high-salt group exhibited higher blood pressure at all timepoints compared with the low-salt group.

**Figure 1:**
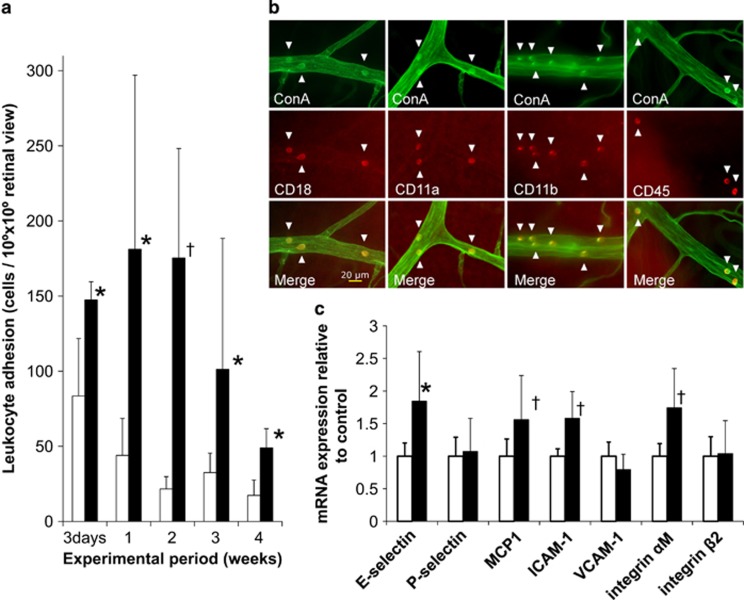
(**a**) Effects of a high salt load on leukocyte adhesion to retinal vessels. The number of leukocytes adhered to retinal vessels was determined using acridine orange fluorescence (see text for details). Open bars represent Dahl S rats fed the low-salt diet, and solid bars represent Dahl S rats fed the high-salt diet. The high-salt diet was given to 4-week-old rats. Rats were tested 3 days after loading and then weekly for 4 weeks. Differences were assessed using the Mann–Whitney U-test with Bonferroni correction. *P<0.01 and ^†^*P*<0.001 *vs.* the low-salt group at each time point. (**b**) Characterization of leukocytes adhered to retinal vessels. Cells were stained with fluorescein isothiocyanate conjugated with ConA (top, green), and anti-CD18, CD11a, CD11b and CD45 antibodies (middle, red). Signals of ConA were consistent with those of the antibodies (bottom, Merge), thereby indicating that the ConA-positive cells detected were leukocytes loaded with CD18, CD11a, CD11b and CD45 antigens. Arrowheads indicate leukocytes adhered to the retinal artery. Scale bar, 20 μm. (**c**) Adhesion molecule mRNA expression in the kidney. Adhesion molecule mRNA expression in the kidney of 7-week-old Dahl S rats was determined 3 weeks after salt loading. Open bars represent Dahl S rats fed the low-salt diet (*n*=5), and solid bars represent Dahl S rats fed the high-salt diet (*n*=5). The mRNA content is expressed relative to glyceraldehyde 3-phosphate dehydrogenase (GAPDH) mRNA in the kidney tissue. Differences were assessed using the Kruskal–Wallis test followed by Mann–Whitney U-test with Bonferroni correction. *P<0.02 and ^†^*P*<0.01 *vs.* Dahl S rats fed the low-salt diet.

The high-salt diet significantly increased leukocyte adhesion to retinal vessels at day 3 when the average blood pressure was 125 mm Hg in the high-salt group and 123 mm Hg in the low-salt group (Figure 1a). In addition to blood pressure elevation with a high salt intake, leukocyte adhesion slightly increased 2 weeks after the salt challenge; however, it declined at weeks 3 and 4, whereas blood pressure still increased. Leukocyte adhesion was higher in the high-salt group than in the low-salt group throughout the experiment, while the total number of peripheral leukocytes and their differentiation did not differ between the two groups.

By contrast, in 4-week-old SHRs, the number of leukocytes adhering to the retinal vessel was lower than in Dahl S rats, and the high salt loading did not increase leukocyte adhesion in SHRs at week 1 (40.9±9.4 *vs.* 36.9±11.4 cells per 10 × 10° retinal view in the high-salt and low-salt groups, respectively).

### Characterization of leukocytes adhering to the retinal vessels

We characterized the adhered nucleated cells using fluorescein isothiocyanate conjugated to ConA lectin. Approximately 95% of the leukocytes stained with ConA were positive for anti-CD18 antibody staining. The cells were also positive for CD11a (90%), CD11b (55%) and CD45 (71%) antibody staining (Figure 1b). Double staining showed that each antibody signal matched the ConA signal of leukocyte adherence to the retinal vessels.

To investigate the expression of adhesion molecules after salt loading, we determined the mRNA expression in renal tissue using real-time PCR. As shown in Figure 1c, the expression levels of e-selectin, ICAM-1, and integrin αM were significantly higher in the high-salt group than in the low-salt group (e-selectin, *P*<0.02, ICAM-1, *P*<0.01, and integrin αM, *P*<0.01).

### Leukocyte adhesion and the renin–angiotensin system

We examined whether the increased leukocyte adhesion in response to a high-salt diet was mediated by the renin–angiotensin system. In the present study, we utilized losartan, an angiotensin II subtype-1 receptor antagonist, to block signal transduction of angiotensin II. Systolic blood pressure was increased in the five experimental groups in a time-dependent manner (Table 2). However, angiotensin II antagonism decreased the rise in blood pressure due to high salt intake as compared with the untreated Dahl S rats fed the high-salt diet. Blood pressure was higher in Dahl S rats treated during the first half (early period) of the experiment than those treated during whole period.

**Figure 2:**
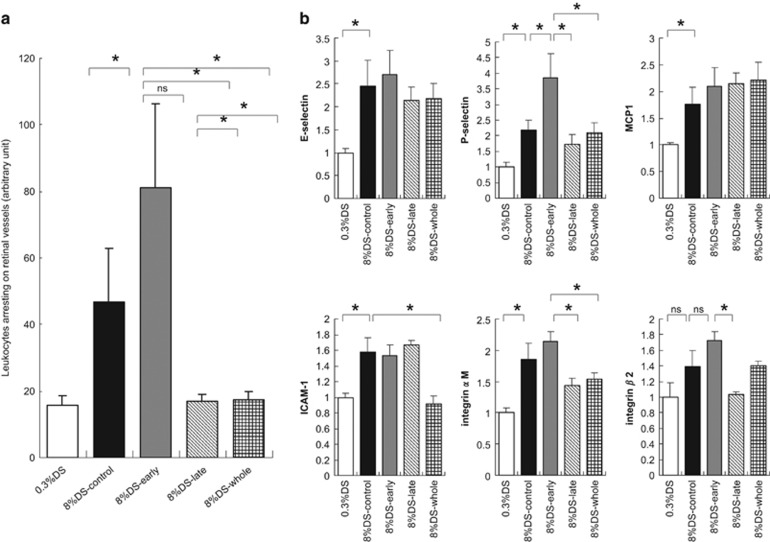
(**a**) Influence of angiotensin receptor antagonism on leukocyte adhesion. Leukocytes adhered to retinal vessels were measured using acridine orange fluorescence (see text for details). Experimental groups: 0.3%DS, Dahl S rats fed a low-salt (0.3%) diet; 8%DS-control, Dahl S rats fed a high-salt (8% NaCl) diet; 8%DS-early, Dahl S rats fed a high-salt diet and treated with losartan for the first 10 days only; 8% DS-late, Dahl S rats fed a high-salt diet and treated with losartan for the last 10 days only; 8%DS-whole, Dahl S rats fed a high-salt diet and treated with losartan throughout the experiment. Differences were assessed using the Kruskal–Wallis test followed by the Mann–Whitney U-test with Bonferroni correction. *P<0.05. (**b**) Determination of mRNA of adhesion molecules in the kidneys. mRNA levels of adhesion molecules in the kidneys were quantitatively determined by real-time PCR as described in the text. Tissue mRNA contents were standardized relative to the content in Dahl S rats fed a low-salt diet alone. Differences were assessed using the Kruskal–Wallis test followed by the Mann–Whitney U-test with Bonferroni correction. *P<0.05.

Losartan treatment significantly decreased the number of adhered leukocytes to the level of that in the low-salt group when administered during the second half (late period) of the experiment (Figure 2a). Losartan treatment during the late period generally decreased the mRNA level of ICAM-1, ICAM-1-related molecules, integrin αM and integrin β2 compared with levels in the untreated high-salt group, whereas the mRNA expression was almost unchanged in Dahl rats treated during the early period (Figure 2b).

### Inhibition of leukocyte adhesion and kidney damage

ICAM-1 and ICAM-1-related adhesion molecules were upregulated, in addition to CD18-laden leukocyte adhesion, in response to the high salt loading. We directly blocked tethering of CD18-loaded leukocytes to ICAM-1 and ICAM-1-related molecules using anti-CD18-specific antibodies.

**Figure 3:**
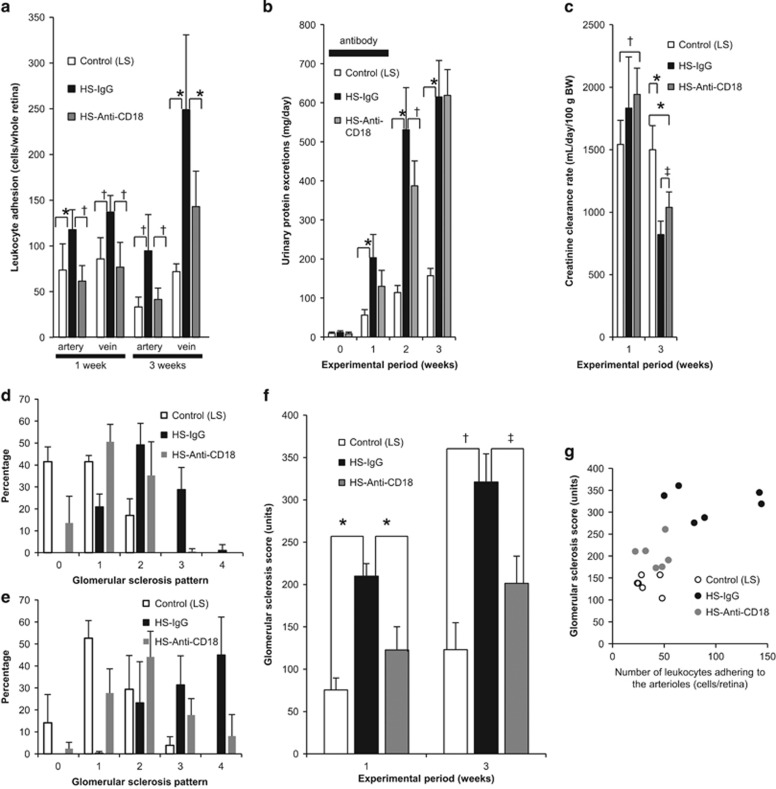
Control (LS) represents Dahl S rats fed a low-salt (0.3% NaCl), HS-IgG represents Dahl S rats fed a high-salt (8% NaCl) diet and given a nonspecific IgG fraction, and HS-Anti-CD18 represents Dahl S rats fed a high-salt diet and given an active anti-CD18-specific antibody. (**a**) Blocking of leukocyte adhesion by anti-CD-specific antibody. We evaluated the effects of anti-CD18 antibody on leukocytes adhered to the arterioles and the smaller veins 1 and 3 weeks after the start of the experiment. Differences were assessed using the Kruskal–Wallis test followed by the Mann–Whitney U-test with Bonferroni correction. *P<0.025, ^†^*P*<0.005. (**b**) Urinary protein excretion. Differences were assessed using the one-way ANOVA followed by post-hoc analysis using the Scheffe test. *P<0.0001, ^†^*P*<0.01. (**c**) Creatinine clearance rate. Differences were assessed using the one-way ANOVA followed by *post-hoc* analysis using the Scheffe test. *P<0.0001, ^†^*P*<0.01, ^‡^*P*<0.05. (**d**) Glomerular sclerosis pattern 1 week after commencement of the experiment. (**e**) Glomerular sclerosis pattern 3 weeks after commencement of the experiment. (**f**) Glomerular sclerosis score. Left and right scales show scores 1 week and 3 weeks after commencement of the experiment, respectively. Differences were assessed using the one-way ANOVA followed by *post-hoc* analysis using the Scheffe test. *P<0.005, ^†^*P*<0.02, ^‡^*P*<0.025. (**g**) Correlation between the number of leukocytes adhering to the arterioles and glomerular sclerosis score 3 weeks after commencement of the experiment. Correlation was performed using simple Pearson correlations. ANOVA, analysis of variance.

The high-salt diet increased the number of CD18-positive leukocytes adhered to the retinal arterioles and smaller veins at weeks 1 and 3 compared with the low-salt diet (Figure 3a). Treatment with anti-CD18 antibodies significantly decreased leukocyte adherence at week 1, and the decrease in leukocyte adhesion was maintained at week 3.

Because anti-CD18 antibodies blocked the ligation, we examined functional or morphological changes in the kidney of Dahl S rats with salt-induced hypertension. Treatment with anti-CD18 antibodies did not influence body weight and urinary sodium excretion at week 1 (Table 3). Administration of anti-CD18 antibody attenuated the increase in proteinuria, and the creatinine clearance rate was significantly increased compared with the HS-IgG group (Figures 3b and c).

We examined morphologically the protection from renal damage resulting from adhesion blocking. As shown in Figures 3d–f, the high-salt diet significantly increased the glomerular sclerosis score; this increase was significantly attenuated by antibody treatment, and the effect was observed until 12 days after treatment cessation (3 weeks after loading). The glomerular sclerosis score was significantly correlated with the number of leukocytes adhering to the arterioles (Figure 3g, *r*=0.62, *P*<0.05). Treatment with anti-CD18 antibodies did not influence body weight but improved urinary sodium excretion at week 3 (Table 3).

## Discussion

The most important finding in the present study was that leukocyte adhesion was increased in response to a high-salt challenge in Dahl S rats. The adhesion quickly increased 3 days after salt loading when systolic blood pressure was within a normotensive range. The mechanism of the rapid leukocyte adherence response was unclear. We demonstrated that the early response of leukocyte adhesion to high-salt intake was not abolished by the angiotensin II subtype-1 receptor antagonist losartan, whereas the later phase of the response was almost completely abolished by the antagonist. These findings suggest that the mechanisms underlying the leukocyte response to high salt intake differed between the early and later phases. At present, we do not have data to explain the mechanism of the angiotensin II-independent events. However, it has been reported that levels of vascular vasodilators, such as prostacyclin and nitric oxide, are reduced in Dahl S rats while those of vasoconstrictors are enhanced.^27^ Since prostacyclin and nitric oxide are potent inhibitors of leukocyte adhesion to endothelial cells, the imbalance between vasodilator and vasoconstrictor substances, as well as an increase in shear stress, may be responsible for the angiotensin II-independent leukocyte adhesion in the early phase.^28, 29, 30, 31^ On the basis of these findings, leukocyte adhesion in response to high salt loading is multifactorial and the role of the renin–angiotensin system becomes apparent along with progression of vascular damage.

Angiotensin II activates nicotinamide adenine dinucleotide phosphate oxidase in leukocytes and thereby increases the amount of radicals. There is considerable evidence suggesting that oxygen radicals have an important role in damaging endothelial cells and cardiac function in advanced heart failure in Dahl S rats.^32, 33, 34^ In the present study, we did not investigate the role of oxygen stress in leukocyte adhesion-related kidney injury. However, we have reported previously that progression of salt-induced hypertension and associated kidney damage is attenuated by oxygen radical scavengers, and immune suppression decreases leukocyte infiltration in periarteries and is associated with attenuation of kidney dysfunction in Dahl S rats.^35, 36, 37^ These findings suggested that, at least in established salt-induced hypertension in Dahl S rats, oxygen radicals are involved in progression of kidney damage. In fact, it has been reported that Dahl S rats are susceptible to angiotensin-induced kidney damage. Thus, it is possible that the rapid increase in leukocyte adhesion in response to a high-salt diet may be related to the high sensitivity of the renin–angiotensin system in Dahl S rats.^14, 15^ Furthermore, we demonstrated that leukocyte adhesion did not increase in response to a high salt load in the rat model of SHR. This rat model is less salt-sensitive than spontaneously hypertensive stroke prone (SHRsp) rats or Dahl S rats. Blood volume and plasma volume were reported to be essentially normal in young SHRs and moderate salt loading has weak pressor effects in very young SHRs.^38, 39^ The increase in leukocyte adhesion in Dahl S rats is related to salt sensitivity, but is not a consequence of the elevation of blood pressure.

Ang II inhibition with losartan in the early stage of salt loading was associated with upregulation of p-selectin. The mechanism was not clear; however, we may address at least two possibilities. Firstly, we have reported that Ang II-mediated intracellular signal transduction also upregulates the regulator of G protein signaling 2 which attenuates intracellular Ang II signal transduction.^40^ A small amount of Ang II in the early stage of salt loading more likely influences regulator of G protein signaling 2 regulation than the signal transduction. Secondly, losartan inhibits Ang II-mediated action through receptor antagonism and it also directly stimulates regulator of G protein signaling 2 biosynthesis, suggesting that Ang II-mediated signal transduction is potently down-regulated by both receptor-mediated and regulator of G protein signaling 2-mediated mechanisms. The withdraw of losartan may provoke upregulation of Ang II signal transduction in the subsequent phase.

The inhibition of ICAM-1 binding to its receptors by specific antibodies decreased urinary protein excretion in Dahl S rats; the decrease continued for 5 days after treatment cessation. Angiotensin II activates nicotinamide adenine dinucleotide phosphate oxidase and increases oxygen radical generation. Oxygen stress influences nuclear factor kappa B-related transcriptional regulation of various bioactive substances.^1^ In the present study, we did not examine the status of oxygen radicals when the rats were challenged with a high-salt diet. However, we reported previously that renal damage in Dahl S rats with hypertension is attenuated by scavengers of oxygen radicals.^14^ It seems probable that the rapid response of leukocyte adhesion to a high-salt diet is mediated by a mechanism involving angiotensin/nicotinamide adenine dinucleotide phosphate and oxygen radicals.

We demonstrated that the inhibition of leukocyte adhesion was associated with the attenuation of kidney damage. We did not directly examine the leukocytes on the vessels of the kidney. However, the blocking antibody indeed reduced leukocyte adherence in the vessels of the retina, and it is presumed that this antibody was effective in the vessels of the kidney. In fact, we demonstrated that the CD18 antigen was expressed in tethered leukocytes and that mRNA of the CD18-binding molecules and ICAM-1 adhesion molecules was also overexpressed in renal tissue. Moreover, leukocyte adhesion was inhibited by the anti-CD18 antibody. Taken together, our results strongly suggest that leukocyte adhesion through ICAM-1 linkage caused renal injury in Dahl S rats.

To assess the adhesive activity of leukocytes in the target organ circulation, we observed leukocytes tethered to the retinal vessels using acridine orange fluoroscopy. Myeloperoxidase activity has been used thus far as a surrogate marker for leukocyte infiltration into the kidney. Recent advances in technology made it possible to investigate the superficial microvasculature in the hydronephrotic kidney induced by bilateral ligation of the ureter^41^ or in the vital kidney using intravital two-photon imaging.^42^ In our studies, however, we investigated morphological injury in glomeruli localized in the deep cortex. Glomeruli in the deep cortex are more susceptible to injury in Dahl S rats as compared with those in the superficial cortex. It might be technically difficult to investigate leukocyte migration *in vivo* in the deep microvasculature.

In relation to this, there has been much evidence that microalbuminuria is associated with cardiovascular disease,^43, 44, 45^ suggesting that microalbuminuria reflects widespread vascular damage.^46, 47, 48^ Several mechanisms have been proposed, including an inflammatory process, but there is not yet sufficient direct evidence to suggest that vasculature in the retina can predict widespread vascular damage; however, recent advances in endothelial cell integrity may suggest that retinal microvasculature may be implicated in predicting widespread damage.^49, 50^

More interestingly, we demonstrated that leukocytes more likely adhered to veins than arteries in response to salt loading. Joussen *et al.*^20^ reported that leukocytes often adhere to post-capillary vessels. The pathophysiological implications remain to be elucidated; however, it is noteworthy that the initiation of vascular damage may occur in post capillary vessels rather than arterioles of the kidney.

Recent studies have demonstrated that leukocyte adhesion in the cerebral circulation is related to the SHR.^6^ Mazor *et al.*^51^ reported that leukocyte depletion significantly attenuated the development of hypertension in salt-loaded Sabra rats. In our study, anti-CD-18 antibody treatment with decreased leukocyte adherence did not influence the blood pressure in Dahl S rats (data not shown). The reason for the discrepancy was not clear. However, in our study we observed an early response of leukocytes to salt loading and the effects blockade using a short-term anti-CD18 antibody infusion on blood pressure and kidney damage. Long-term inhibition would have provided clearer evidence of anti-hypertension and protection of kidney function.

Finally, in general, high-salt challenges decrease the activity of the renin–angiotensin system. In this study, we demonstrated the involvement of the renin–angiotensin system in leukocyte adhesion and the related organ damage. In this context, we have reported that the renin–angiotensin system in Dahl S rats is not sufficiently suppressed by a high-salt challenge, as compared with a low-salt diet,^52^ and this strain exhibits a higher response to angiotensin II stimulation.^24, 53^ This may be caused by a depressed vasodilator system or the impaired regulator of Gαq signaling-2 (RGS-2) that downregulates intracellular angiotensin II signal transduction.^54^

In conclusion, we demonstrated that a high-salt challenge increases leukocyte adhesion to retinal vessels in Dahl S rats, probably through the renin–angiotensin axis. Furthermore, inhibiting ICAM-1 binding to its receptors resulted in decreased leukocyte adhesion to retinal vessels, and this was associated with the attenuation of kidney damage. This mechanism may lead to new strategies for renal protection during salt-induced hypertension.

## Figures and Tables

**Table 1:** Systolic blood pressure in experiment 1

*Group*	*Day 3*	*Week 1*	*Week 2*	*Week 3*	*Week 4*
Low-salt	123±2 (18)	134±6 (15)	147±5 (12)	152±4 (9)	155±5 (6)
High-salt	125±2* (19)	151±9** (16)	188±22** (13)	208±12** (10)	216±11** (6)

Abbreviation: ANOVA, analysis of variance.

Low-salt, Dahl S rats fed a low-salt (0.3%) diet; high-salt, Dahl S rats fed a high-salt (8% NaCl) diet. Differences were assessed by one-way ANOVA. Numbers in parentheses represent the numbers of rats.

*P<0.05, **P<0.001 *vs.* respective values in low-salt group.

**Table 2:** Systolic blood pressure in experiment 2

*Group*	*Start*	*Week 1*	*Week 2*	*Week 3*
0.3%DS	138±2	145±2*	152±1*	156±2*
8%DS-control	139±1	176±2	204±2	222±4
8%DS-early	138±2	174±2	203±3	208±2*
8%DS-late	139±1	174±2	198±3*^,†^	204±2*^,†^
8%DS-whole	138±2	177±2	199±1*^,†^	207±3*

Abbreviation: ANOVA, analysis of variance.

Experimental groups: 0.3%DS, Dahl S rats fed a low-salt (0.3%) diet; 8%DS-control, Dahl S rats fed a high-salt (8% NaCl) diet; 8%DS-early, Dahl S rats fed a high-salt diet and treated with losartan for the first 10 days only; 8%DS-late, Dahl S rats fed a high-salt diet and treated with losartan for the last 10 days only; 8%DS-whole, Dahl S rats fed a high-salt diet and treated with losartan throughout the experiment. There were five rats per group. Differences were assessed by one-way ANOVA followed by post-hoc analysis using the Scheffe test.

*P<0.001 *vs.* respective values in 8% DS-control group, ^†^*P*<0.05 *vs.* respective values in 8% DS-early group.

**Table 3:** Body weight and urinary sodium excretion

*Body weight (g)*	*Start*	*Week 1*	*Week 2*	*Week 3*
Control (LS)	105±2.2	158±1.7	207±2.1	257±2.1
HS-IgG	101±1.5	155±2.6	202±3.8	223±4.2*
HS-Anti-CD18	102±1.7	153±2.1	198±2.8	235±4.3^†^
*Urinary sodium excretion (mEq per day per 100 g body weight)*				
Control (LS)	0.26±0.09	0.33±0.05	0.35±0.06	0.32±0.03
HS-IgG	0.34±0.16	16.22±1.53*	13.70±0.98*	6.97±3.41*
HS-Anti-CD18	0.27±0.09	15.81±0.77*	13.98±0.91*	12.03±0.70*^,‡^

Abbreviation: ANOVA, analysis of variance.

The difference was assessed by one-way ANOVA followed by *post-hoc* analysis using the Scheffe test. Values are presented as mean±s.d.

Control (LS), Dahl S rats fed a low-salt (0.3% NaCl); HS-IgG, Dahl S rats fed a high-salt (8% NaCl) diet and given a nonspecific IgG fraction; HS-Anti-CD18, Dahl S rats fed a high-salt diet and given an active anti-CD18-specific antibody.

*P<0.0001, ^†^*P*<0.005 *vs.* control (LS), ^‡^*P*<0.005 *vs.* HS-IgG. There was no other difference between the HS-IgG and HS-Anti-CD18 groups.
