# Supplementary material for: A high-salt diet enhances leukocyte adhesion in association with kidney injury in young dahl salt-sensitive rats
Source: Hypertens Res. 2017 Mar 16;40(11):912–20. doi: 10.1038/hr.2017.31 (PMC5675900; doi:10.1038/hr.2017.31)
Supplement: Supplementary Table 1 [file hr201731x1.doc]

Supplementary 1 : Design of the primers/probes

| Adhesion molecules | primers/probes | |
| --- | --- | --- |
| E-selectin | forward | TCTAAGATGCGAGCAATCAGG |
|  | reverse | CAGGATTCTGCAGACAGTTCA |
|  | probe | #18 |
| P-selectin | forward | AATCCCCCGCAGTGTAAAG |
|  | reverse | GGGTGTGTACAGTCCATGGTT |
|  | probe | #116 |
| MCP1 | forward | AGCATCCACGTGCTGTCTC |
|  | reverse | GATCATCTTGCCAGTGAATGAGT |
|  | probe | #62 |
| ICAM-1 | forward | TTCTGCCACCATCACTGTGT |
|  | reverse | AGCGCAGGATGAGGTTCTT |
|  | probe | #74 |
| Vascular cell adhesion molecule-1 | forward | CAAATGGAGTCTGAACCCAAA |
|  | reverse | GGTTCTTTCGGAGCAACG |
|  | probe | #13 |
| integrin αM | forward | ACTCTGATGCCTCCCTTGG |
|  | reverse | CCTGGACACGTTGTTCTCAC |
|  | probe | #68 |
| integrin β2 | forward | tccacaaaaagtgacccttaact |
|  | reverse | CGTCGGAAAGTCACATTGAA |
|  | probe | #66 |
| GAPDH | forward | AATGTATCCGTTGTGGATCTGA |
|  | reverse | GCTTCACCACCTTCTTGATGT |
|  | probe | #80 |
